# Plasmid-Encoded Pgp5 Is a Significant Contributor to *Chlamydia muridarum* Induction of Hydrosalpinx

**DOI:** 10.1371/journal.pone.0124840

**Published:** 2015-04-27

**Authors:** Yumeng Huang, Qi Zhang, Zhangsheng Yang, Turner Conrad, Yuanjun Liu, Guangming Zhong

**Affiliations:** 1 Department of Microbiology & Immunology, University of Texas Health Science Center at San Antonio, 7703 Floyd Curl Drive, San Antonio, Texas, 78229, United States of America; 2 Department of Dermatology, 2^nd^ Xiangya Hospital, Xiangya School of Medicine, Central South University of China, Changsha, Hunan 410013, P. R. China; 3 Department of Obstetrics and Gynecology, 3^rd^ Xiangya Hospital, Xiangya School of Medicine, Central South University of China, Changsha, Hunan 410013, P. R. China; 4 Department of Dermatovenereology, Tianjin Medical University General Hospital, 154 Anshan Rd., Tianjin 300052, P. R. China; Midwestern University, UNITED STATES

## Abstract

We have previously shown that the plasmid-encoded Pgp3 is a major virulence factor for *C*. *muridarum* induction of hydrosalpinx. We now report that Pgp5 also plays a significant role in the development of hydrosalpinx following *C*. *muridarum* induction. Pgp5 deficiency was introduced via either in-frame deletion (CM-Δpgp5) or premature stop codon installation (CM-pgp5S). Mice infected with either CM-Δpgp5 or CM-pgp5S developed hydrosalpinges at significantly reduced levels with an incidence rate of <40% and a mean severity score of 2 or less. In contrast, 80% or more mice developed hydrosalpinx with a severity score of >3 when mice were infected with Pgp5-sufficient *C*. *muridarum* (plasmid-competent wild type or plasmid-free *C*. *muridarum* transformed with a full plasmid or depleted of pgp7 gene). The attenuated pathogenicity of the Pgp5-deficient *C*. *muridarum* correlated with a significantly reduced level of ascending infection in the oviduct tissue despite the similar overall shedding courses between mice infected with Pgp5-deficeint versus sufficient *C*. *muridarum*. Furthermore, in the oviducts of mice infected with Pgp5-deficient *C*. *muridarum*, significantly lower levels of inflammatory cell infiltration and cytokine production were detected. Thus, Pgp5 is a significant plasmid-encoded virulence factor for *C*. *muridarum* pathogenicity in the upper genital tract.

## Introduction

Induction of hydrosalpinx in the mouse upper genital tract by *Chlamydia muridarum* infection in the lower genital tract has been used for investigating the mechanisms of chlamydial pathogenesis and immunity [1,2,3,4,5,6,7]. Studies based on this mouse model have led to the hypothesis that both adequate ascension to and induction of the appropriate inflammatory responses in the upper genital tract are necessary for hydrosalpinx development [7,8,9,10,11]. Thus, searching for the chlamydial virulence factors required for *C*. *muridarum* ascension and induction of oviduct inflammation has become a hot topic under extensive investigation.

Hydrosalpinx induction by *C*. *muridarum* in some strains of mice is known to depend on the cryptic plasmid [6,7] that encodes 8 genes designated as *pgp*1-8 [12,13,14,15]. This highly conserved plasmid is also important in *C*. *trachomatis* pathogenesis [16,17]. A transformation approach has been used for characterizing the chlamydial plasmid [18,19,20,21,22,23,24,25]. The genes coding for Pgp3, 4, 5 and 7 can be deleted from both *C*. *trachomatis* serovar L2 [19,20] and *C*. *muridarum* [26] plasmids. Pgp4 has been shown to positively regulate many plasmid-encoded and-dependent chromosomal genes while Pgp5 is capable of negatively regulating some plasmid-dependent chromosomal genes. However, Pgp3 or 7 does not significantly affect the expression of the other chlamydial genes. Despite the extensive *in vitro* characterization, the roles of these plasmid-encoded Pgps in chlamydial pathogenesis must be evaluated in animal models. Using the mouse model, we recently reported that the *C*. *muridarum* transformants with either in-frame deletion of or premature termination codon insertion in *pgp3* failed to induce any significant hydrosalpinx following an intravaginal infection [27]. The attenuated pathogenicity of the Pgp3-deficient *C*. *muridarum* further correlated with a rapid decrease in chlamydial survival in the lower genital tract and reduced ascension to the upper genital tract. The Pgp3-deficient organisms were also less invasive when delivered directly to the oviduct. These observations have demonstrated that plasmid-encoded Pgp3 is required for *C*. *muridarum* survival in the mouse genital tract and represents a major virulence factor for *C*. *muridarum* pathogenesis in mice. This conclusion is supported by the observation that a *C*. *trachomatis* L2 organism with *pgp3* gene deletion displayed a significantly reduced infectivity and pathogenicity in the mouse genital tract [28].

In the current study, we investigated the role of Pgp5, a putative negative regulator, in *C*. *muridarum* pathogenesis. We found that Pgp5 deficiency in the form of either in-frame deletion or premature stop codon installation significantly reduced the ability of *C*. *muridarum* to induce hydrosalpinx. The reduced pathogenicity correlated with a significant decrease in ascending infection and inflammatory infiltration and cytokine production in the oviduct tissue. Thus, we have identified Pgp5 as another plasmid-encoded virulence factor in *C*. *muridarum* induction of hydrosalpinx.

## Materials and Methods

### 1. Chlamydial organisms and infection

The wild type (wt) cryptic plasmid-containing *Chlamydia muridarum* (Nigg) organisms were propagated, purified, aliquoted and stored as described previously [29]. HeLa cells (human cervical epithelial carcinoma cells, cat# CCL2) used in the current study were purchased from ATCC (Manassas, VA). Chlamydial infection in cell culture was carried out as described previously [7,30] with the modification that a combination of anti-Pgp3 and anti-chlamydial organism antibody labeling approach was used for identifying plaques free of plasmid (unpublished data). Both the initial plasmid-free and the subsequent transformants were plaque-cloned using a standard plaque assay as described previously [26,31]. The following *C*. *muridarum* transformants were used in the current study, including CMUT3 transformed with the intact plasmid pGFP::CM (CM-pGFP::CM) or pGFP::CM with a premature stop codon in pgp3 (CM-pgp3S), 4 (CM-pgp4S) or 5 (CM-pgp5S) or deletion of pgp5 (CM-Δpgp5) or 7 (CM-Δpgp7). These transformants were produced and plaque-purified as described previously [26,27].

### 2. Plaque-forming assay and plaque size determination

Stocks of *Chlamydia muridarum* organisms were inoculated onto confluent monolayers of McCoy cells in six-well or 24-well tissue culture plates and centrifuged at 1,200 rpm for 1 h at room temperature (RT). The inoculum was then removed and replaced with overlay medium (1× Dulbecco's modified Eagle's medium, 10% fetal bovine serum, 1μg/ml cycloheximide, and a final concentration of 0.55% of agarose). The cells were then incubated at 37°C in an atmosphere of 5% CO_2_ for 5 days to allow plaques to form. The solid overlay was removed, and the monolayers were fixed in methanol and then stained with 0.03% Neutral Red in phosphate-buffered saline for 1h at RT. The visualized plaques in the six-well plate were scanned and images were saved in Adobe Photoshop. The areas of plaques were measured using the custom MATLAB program plaqueDetector. Briefly, this software automatically detects cell culture plate wells within an input image, automatically detects plaques within each well, discards plaques in close proximity to the well edge (default is within 10% of well diameter), and normalizes the plaque areas to the area of their encompassing well to allow for comparison of images taken at varying heights. Well and plaque sensitivity and edge threshold settings were tailored to each image to reduce the occurrence of false positive and false negative plaques. The plaqueDetector can be accessed freely at http://www.mathworks.com/matlabcentral/fileexchange/48860-plaque-detector.

### 3. Mouse infection and live organism recovery from mouse vaginal/cervical swabs and tissue homogenates

Various *C*. *muridarum* organisms including CM(wt), CMUT3(pf), CM-pGFP::CM, CM-pgp5S, CM-Δpgp5 and CM-Δpgp7 were used to infect female C3H/HeJ mice (6 to 7 week old, Jackson Laboratories, Inc., Bar Harbor, Maine) intravaginally with 2 X 10^5^ inclusion-forming units (IFUs). Five days prior to the infection, each mouse was injected with 2.5 mg medroxyprogesterone (Depo-Provera; Pharmacia Upjohn, Kalamazoo, MI) subcutaneously to increase mouse susceptibility to the infections. After infection, mice were monitored for vaginal live organism shedding and by day 60 or different days (as indicated in individual experiments) after infection, mice were sacrificed for observing genital tract pathologies or titrating *C*. *muridarum* live organisms in different sections of the mouse genital tract. The animal experiments were carried out in accordance with the recommendations in the Guide for the Care and Use of Laboratory Animals of the National Institutes of Health. The protocol was approved by the Committee on the Ethics of Laboratory Animal Experiments of the University of Texas Health Science Center at San Antonio.

For monitoring live organism shedding from swab samples, vaginal/cervical swabs were taken every 3 to 4 days for the first week and weekly thereafter until negative shedding for 2 consecutive time points. To quantitate live chlamydial organisms, each swab was soaked in 0.5 ml of SPG and vortexed with glass beads, and the chlamydial organisms released into the supernatants were titrated on HeLa cell monolayers in duplicate as described previously [32]. The total number of IFUs per swab was calculated and converted into log_10_, and the log_10_ IFUs were used to calculate the mean and standard deviation at each time point.

To monitor ascending infection, mice infected intravaginally in parallel experiments were sacrificed on day 10 after infection. The entire genital tracts were sterilely harvested, and each tract was divided into 3 portions including vagina/cervix (VC), uterine/uterine horn (UH) and oviduct/ovary (OV). VC was defined as lower genital tract (LGT) while both UH & OV as upper genital tract (UGT). Each tissue portion was homogenized in 0.3ml cold SPG using a 2ml tissue grinder (cat# K885300-0002, Fisher scientific, Pittsburg, PA). After brief sonication and centrifugation at 3000 x rpm for 5min to pellet large debris, the supernatants were titrated for live *C*. *muridarum* organisms on HeLa cells as described above. The results were expressed as log10 IFUs per tissue section homogenate.

### 4. Genital tract pathology

To evaluate genital tract tissue pathology, mice were sacrificed 60 days after intravaginal infection as described above. Before removing the genital tract tissues from the mice, in situ gross examination was performed under a stereoscope (Olympus, Center Valley, PA) for evidence of hydrosalpinx formation and any other gross abnormalities. The genital tract tissues were then isolated in their entirety from the vagina to the ovary and laid on a blue sheet for acquisition of digitized images. The oviduct hydrosalpinges were visually scored based on their dilation sizes using a scoring system as described previously [11]: no oviduct dilation or swelling found with a stereoscope inspection was defined as no hydrosalpinx and assigned a score of zero (0); hydrosalpinx was only visible after amplification, 1; hydrosalpinx was clearly visible with naked eye but the size was smaller than that of ovary,2; size of hydrosalpinx was similar to that of ovary (3) or larger than ovary (4). Both the incidence and severity scores of oviduct hydrosalpinx were analyzed for statistical differences between mice infected with different *C*. *muridarum* isolates.

For histological scoring, the excised mouse genital tract tissues, after photographing, were fixed in 10% neutral formalin and embedded in paraffin and serially sectioned longitudinally (with 5 μm/each section). Efforts were made to include cervix, both uterine horns and oviducts as well as lumenal structures of each tissue in each section. The sections were stained with hematoxylin and eosin (H&E) as described elsewhere [2]. The H&E stained sections were scored for severity of inflammation and pathologies based on the modified schemes established previously [2,33]. Scoring for dilatation of oviduct was as follows: 0, no significant dilatation; 1, mild dilatation of a single cross section; 2, one to three dilated cross sections; 3, more than three dilated cross sections; and 4, confluent pronounced dilation. Inflammatory cell infiltrates were scored as follows: 0, no significant infiltration; 1, infiltration at a single focus; 2, infiltration at two to four foci; 3, infiltration at more than four foci; and 4, confluent infiltration. Scores from both sides of the oviducts were added to represent the oviduct pathology for a given mouse, and the individual mouse scores were calculated into medians for each group. Thus, oviduct lumenal dilation scores and inflammation scores represent microscopic observations. Together with gross pathology parameters of hydrosalpinx incidence and severity, the combination of the 4 parameters allowed us to more accurately describe the oviduct pathology. The researchers who scored the pathology were blinded to the experimental conditions/groups.

### 5. Multiplex array for profiling cytokines in oviduct tissue

Oviduct/ovary tissues were harvested from mice infected with CM-pGFP::CM (n = 5) or CM-pgp5S (n = 5) on day 10 after intravaginal inoculation with *C*. *muridarum* for generating homogenates as described previously [33,34]. The homogenates were used for simultaneous measurements of multiple mouse cytokines using a multiplex bead array assay as described previously [35]. All cytokines are expressed in mean pg/mL plus/minus standard deviation. The means from the two mouse strains were used for calculating ratio and statistics analysis.

### 6. Statistics analyses

Quantitative data including plaque sizes and cytokine concentrations were analyzed using Student’s *t*-test while all semi-quantitative data including the pathology scores and the area under curve (AUC) for comparing the overall IFU shedding courses were analyzed using Wilcoxon rank sum. The number of live organisms as IFUs (inclusion forming units) recovered from tissue homogenates or cell cultures was analyzed with Kruskal-Wallis. All qualitative data including incidence rates were analyzed using Fisher’s Exact.

## Results

### 1. Pgp5-deficient *C*. *muridarum* is significantly attenuated in the mouse upper genital tract

**Fig 1:**
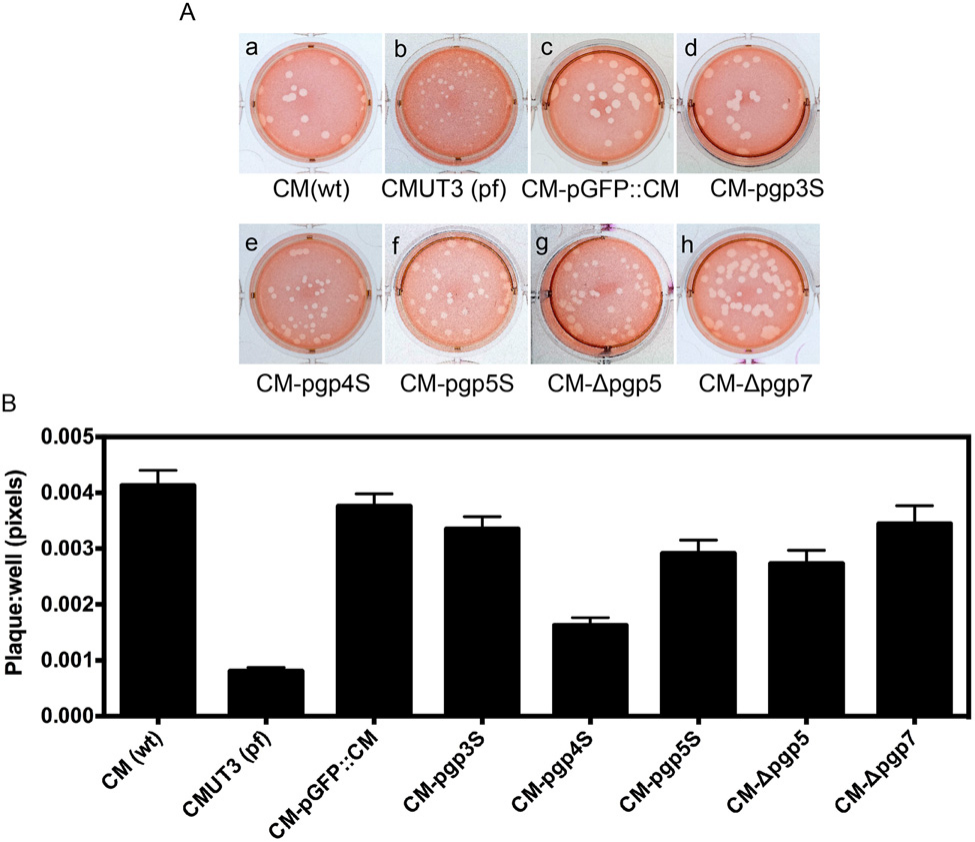
Comparison of plaque sizes among *Chlamydia muridarum* transformants. (A) The following *C*. *muridarum* organisms were inoculated onto McCoy monolayers grown in 6-well plate: Wild type *C*. *muridarum* [CM(wt), image a], plasmid-free *C*. *muridarum* [CMUT3 (pf), b], CMUT3 transformed with the intact plasmid pGFP::CM (CM-pGFP::CM, c) or the pGFP::CM with a premature stop codon in pgp3 (CM-pgp3S, d), 4 (CM-pgp4S, e) or 5 (CM-pgp5S, f) or deletion of pgp5 (CM-Δpgp5, g) or 7 (CM-Δpgp7, h). The cultures were allowed to grow in 0.55% of argarose-containing medium for 5 days before neutral red staining and picture taking. (B) For quantitating the plaque sizes, the stained plates were scanned and the plaque sizes were measured in pixels using the customer-designed software PlaqueDetector. All plaque sizes were normalized using the areas of the corresponding wells so that plaque areas from different wells became comparable. The mean areas and standard deviations were used for comparing between different groups. Note that the plasmid-free CMUT3 and CM-pgp4S produced significantly smaller plaques (p<0.01, Student *t*-test).

**Fig 2:**
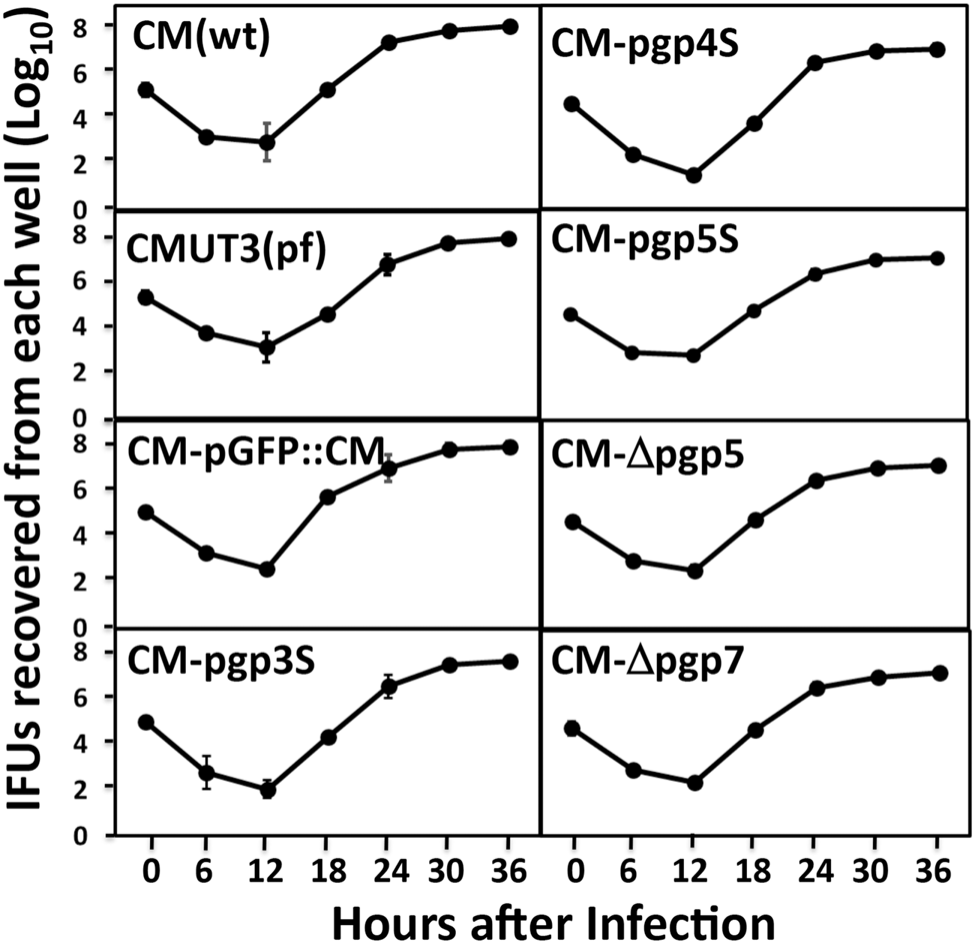
Comparison of the primary growth curves among *Chlamydia muridarum* transformants. All *C*. *muridarum* organisms were inoculated onto McCoy monolayers grown in 24-well plates at an MOI of 0.5 (to avoid over infection). The infected cultures were harvested at 6, 12, 18, 24, 30 & 36 hours after infection as shown along the X-axis for titrating the number of live organisms on fresh monolayers of HeLa cells. The number of live organisms recovered from each culture at each time point was calculated into progeny IFU (inclusion forming unit) per culture as listed along the Y-axis in Log scale. The experiment was repeated 3 three times with duplicate in each. There were no significant differences in the live organism yields between any two organisms at any time points measured (Krustal-Wallis).

Plaques formed by plasmid-deficient *C*. *muridarum* are known to be significantly smaller than those by plasmid-competent *C*. *muridarum* [30]. We used a plaque assay to evaluate whether deficiency in the plasmid-encoded Pgp5 can affect the *in vitro* growth of *C*. *muridarum* (Fig 1). As expected, the plasmid-free CMUT3 produced significantly smaller plaques. The plaque sizes from the Pgp4-deficient *C*. *muridarum* (CM-pgp4S) were also smaller than those from the wild type (wt) *C*. *muridarum* or intact plasmid-transformed *C*. *muridarum* (CM-pGFP::CM). However, the plaque sizes of the *C*. *muridarum* organisms deficient in Pgp5, with either a premature stop codon in the pgp5 gene (CM-pgp5S) or in-frame deletion of pgp5 (CM-Δpgp5), were similar to those of CM(wt) or CM-pGFP::CM. This was also true for CM-pgp3S and CM-Δpgp7. Since the plaque sizes were measured 5 days after infection, which reflected the accumulative results of multiple generations of chlamydial inclusion bursts, we further compared the one-step growth curves among these organisms (Fig 2). In this experiment, all organisms were used to infect McCoy monolayers with the assistance of DEAE pretreatment and centrifugation. The live organisms were recovered from each culture (well) at various time points after infection. We found that all organisms displayed similar growth curves along the entire infection course. Although each well was inoculated with 2 X 10^5^ IFUs at time zero, the number of live organisms recovered at 6h and 12h after infection dropped to 100 to 1000 (significantly below the inoculation size) because of the differentiation into the non-infectious reticulate bodies (RBs) by most of the infectious elementary bodies (EBs). By 18h after infection, the number of live organisms rose very quickly and peaked by 24h to 30h, reaching up to 1 x 10^8^ IFUs (~500 folds of the original inoculum). All organisms regardless of their genotypes followed the same growth kinetics. There were no significant differences in the live organism yields between any two organisms at any time points measured. Thus, all *C*. *muridarum* organisms used in the current study shared similar intracellular growth kinetics. The difference in plaque size between the wild type and plasmid-free *C*. *muridarum* organisms was probably due to the different entry and/or exiting efficiencies between these organisms. Most relevantly (to the current study), the deficiency in Pgp5 did not significantly affect the overall chlamydial growth in cell culture at all.

**Fig 3:**
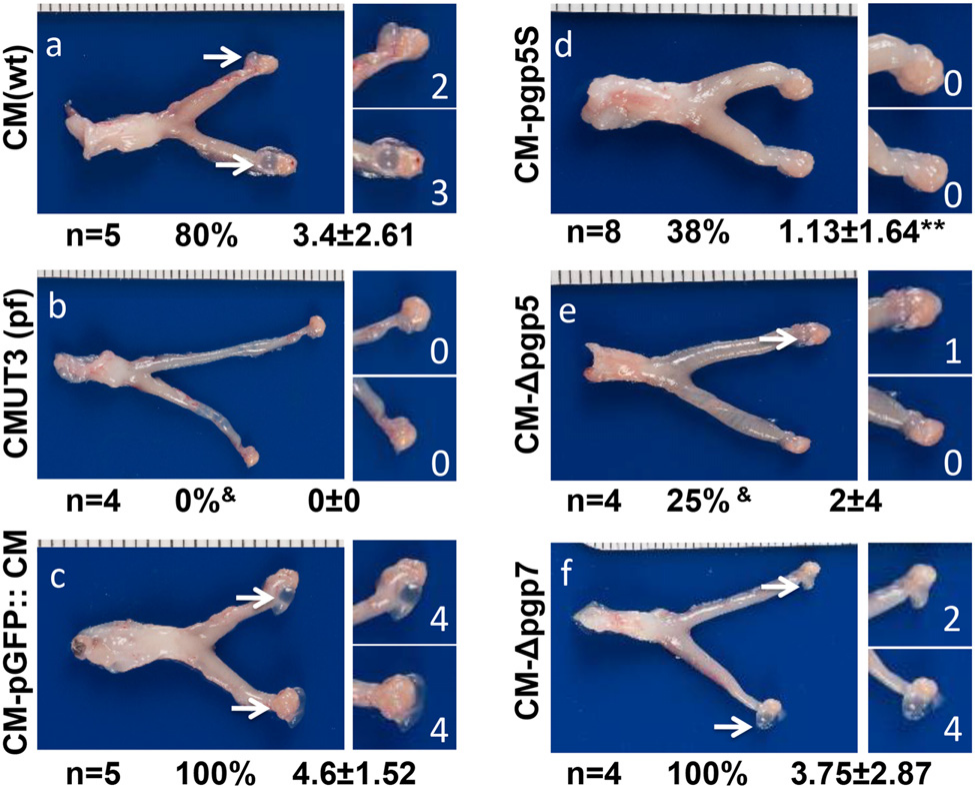
Effect of Pgp5 deficiency on the development of hydrosalpinx following *C*. *muridarum* infection. The wild type (wt) *C*. *muridarum* (CM, panel a), plasmid-free (pf) CMUT3 (b) or CMUT3 transformants (CM-pGFP::CM, panel c; CM-pgp5S, d; CM-Δpgp5, e; CM-Δpgp7, f) were used to intravaginally infect C3H/HeJ mice with an infection dose of 2x10^5^ IFUs. Sixty days after infection, all mice were sacrificed for observing gross appearance of the upper genital tracts. One representative image of the entire genital tract from each group is presented with the vagina/cervix at the left and the oviduct/ovary at the right sides. The total number of mice (n), the incidence (%) and severity (x ± SD) of hydrosalpinges in each group are listed under the corresponding image. Note that the CM(wt) and CM-pGFP::CM induced high incidence of severe hydrosalpinx while the mice infected by CM-pgp5S, CM-Δpgp5 or CMUT3 displayed significant reduced hydrosalpinx. ^&^P<0.05 or ^&&^P<0.01 (Fisher’s Exact); *P<0.05, **P<0.01 (Wilcoxon rank-sum).

**Fig 4:**
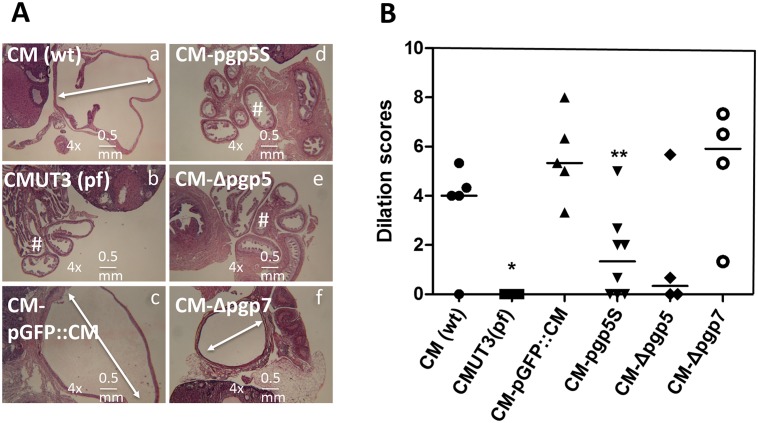
Microscopic observation of oviduct dilation induced by Pgp5-deficient *C*. *muridarum*. The oviduct tissues of mice infected with *C*. *muridarum* organisms as described in Fig 2 legend were harvested and subjected to H&E staining for microscopic evaluation of oviduct dilation. (A) Representative images from each group taken under a 4X objective lens. Dilated oviducts are indicated with white lines with arrowheads at both ends while normal oviducts with a “#” sign. The horizontal bar at the right bottom of each image represents a physical distance of 0.5mm. (B) Severity of lumenal dilation was scored as described in Materials and Methods and listed along the Y- axis [solid circle for CM(wt), star for CMUT3(pf), solid upright triangle for CM-pGFP::CM, solid upside down triangle for CM-pgp5S, solid diamond for CM-Δpgp5 and open circle for CM-Δpgp7). Note that mice infected with CM-pgp5S or CM-Δpgp5 but not CM-Δpgp7 developed significantly lower levels of oviduct lumenal dilation. *p<0.05 (Wilcoxon rank-sum test, all groups were compared against the full plasmid-complemented CMUT3 group, CM-pGFP::CM).

We then evaluated the effect of the Pgp5 deficiency on *C*. *muridarum* induction of hydrosalpinx in mice (Fig 3). The plasmid-free CMUT3 failed to induce any hydrosalpinx while the plasmid-competent CM(wt) or CM-pGFP::CM induced 80% or more mice to develop severe hydrosalpinx with a severity score of 3.4 or greater, which is consistent with previous observations carried out in C3H/HeJ mice [7,27]. Interestingly, deficiency in Pgp5 but not Pgp7 significantly reduced the ability of *C*. *muridarum* to induce hydrosalpinx. The CM-pgp5S- and CM-Δpgp5-infected mouse groups developed hydrosalpinx at incidence rates of 38% and 25% with severity scores of 1.13 and 2 respectively. However, 100% of the CM-Δpgp7-infected mice developed hydrosalpinx with a severity score of 3.75. The hydrosalpinx incidence rate and severity observed with naked eye were also confirmed under microscopy (Fig 4), which revealed significant oviduct dilation in mice infected with CM(wt), CM-pGFP::CM or CM-dpgp7 but not CMUT3, CM-pgp5S or CM-Δpgp5. Thus, we have demonstrated that Pgp5-deficiency significantly reduced *C*. *muridarum* pathogenicity in the mouse genital tract without affecting the *in vitro* growth of *C*. *muridarum*.

### 2. Pgp5-deficient *C*. *muridarum* is less efficient in ascending to the mouse oviduct

**Fig 5:**
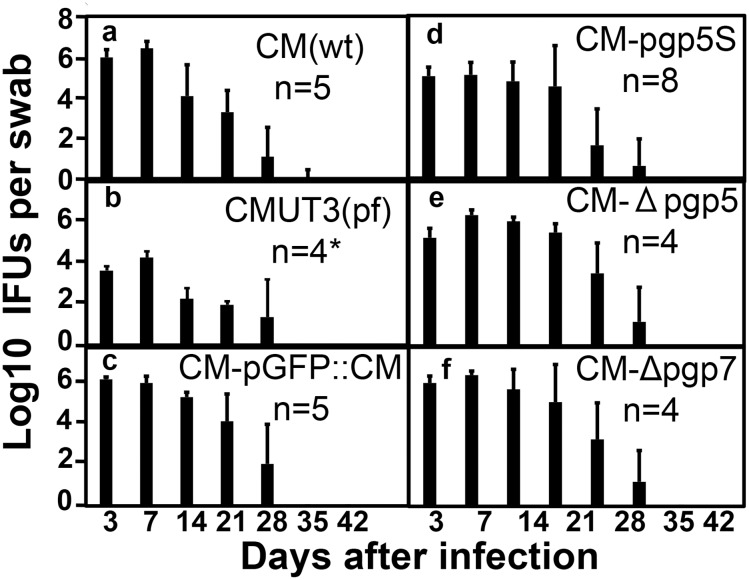
Live organism shedding from mouse lower genital tract following infection with Pgp5-deficient *C*. *muridarum*. The C3H/Hej mice were intravaginally infected with *C*. *muridarum* organisms as described in Fig 2 legend. On different days after infection as shown along the X-axis, vaginal swabs were taken for titrating live organisms on HeLa cell monolayers. The live organisms recovered from each swab are expressed as Log_10_ IFUs along the Y-axis. Note that mice infected with the plasmid free CMUT3 but not other groups displayed a significantly reduced shedding course than the group infected with CM-pGFP::CM. *p<0.05 (Wilcoxon rank-sum test).

**Fig 6:**
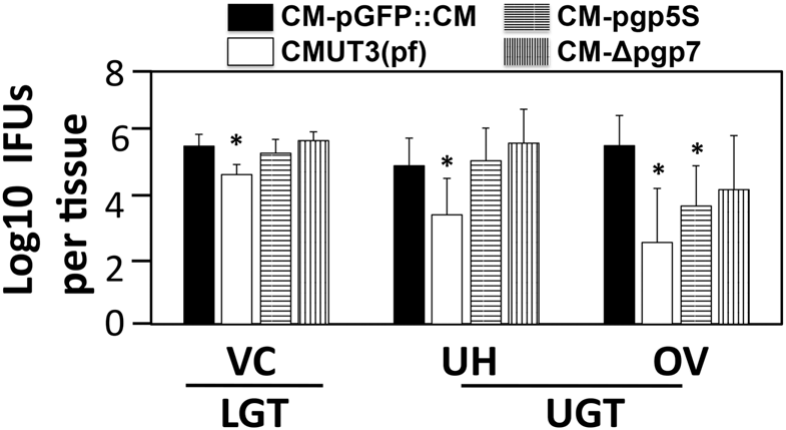
Live chlamydial organism recovery from mouse genital tract tissues following infection with Pgp5-deficient *C*. *muridarum*. C3H/HeJ mice intravaginally infected with *C*. *muridarum* organisms as described in Fig 2 legend [solid bar for CM-pGFP::CM; open bar for CMUT3(pf), horizontally hatched bar for CM-pgp5S and vertically hatched bar for CM-Δpgp7, n = 5 for each group) were sacrificed on days 10 after infection. The entire genital tract tissue was harvested from each mouse and divided into the lower genital tract (LGT) vagina/cervix (VC) and the upper genital tract (UGT) uterus/uterine horn (UH) and oviduct/ovary (OV) sections as listed along the X-axis. Each tissue section was homogenized for titrating live *C*. *muridarum* organisms. The log_10_ IFUs were used to calculate mean and SD for each group as displayed along the Y-axis. Note that the number of live organisms recovered from the oviduct/ovary (OV) homogenates of mice infected with either CMUT3(pf) or CM-pgp5S but not CM-Δpgp7 was significantly lower than that from the CM-pGFP::CM group. * p<0.05 (Kruskal-Wallis).

We further compared the infection courses of mice infected with different *C*. *muridarum* transformants (Fig 5). We found that the lengths of live organism shedding were similar among all mouse groups regardless of the strains of *C*. *muridarum* used for infection. However, the overall level of shedding from mice infected with the plasmid-free *C*. *muridarum* CMUT3 was significantly lower, which is consistent with previous reports [7,27]. Nevertheless, the overall levels of live organism shedding were not significantly altered by either Pgp5 or Pgp7 deficiencies, suggesting that neither Pgp5 nor Pgp7 is required for maintaining the lower genital tract infection of *C*. *muridarum*. Since adequate ascension of live organisms into the oviduct is known to be required for hydrosalpinx induction by *C*. *muridarum* [7,9], we next assessed the role of Pgp5 in *C*. *muridarum* ascension (Fig 6). On day 10 after intravaginal infection, the number of live organisms recovered from the lower genital tract (LGT), vagina/cervix (VC) or the upper genital tract (UGT) uterus/uterine horn (UH) or oviduct/ovary (OV) tissue homogenates of mice infected with the plasmid-free CMUT3 was significantly lower than that of the mice infected with CM-pGFP::CM. However, the Pgp5-deficient (CM-pgp5S) *C*. *muridarum* developed a significantly reduced level of live *C*. *muridarum* organisms only in OV but not any other sections of the genital tract, suggesting that Pgp5 may play an important role in *C*. *muridarum* ascension into or survival in the oviduct tissue. The recovery of CM-Δpgp7 was similar to that of CM-pGFP::CM throughout the genital tract.

### 3. Pgp5-deficient *C*. *muridarum* is less inflammatory in the mouse oviduct

**Fig 7:**
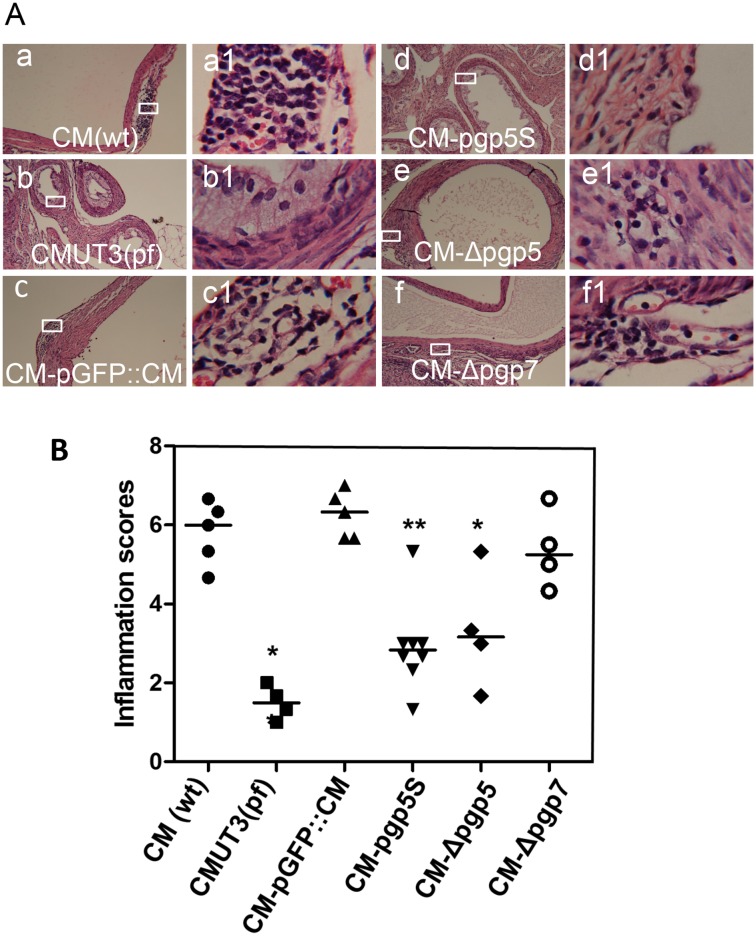
Oviduct inflammatory infiltration induced by Pgp5-deficient *C*. *muridarum*. The oviduct tissues of mice infected with *C*. *muridarum* organisms as described in Fig 2 legend were harvested and subjected to H&E staining for microscopic evaluation of inflammatory infiltration. (A) Representative images from each group taken under 4X (left, panels a-f) and 100X (right, panels a1-f1) objective lens. White rectangles in the 4X lens images indicated the same areas from which the right images were taken under 100X lens. (B) The severity of inflammatory infiltration in oviduct tissue was scored as described in Materials and Methods and listed along the Y- axis [solid circle for CM(wt), star for CMUT3(pf), solid upright triangle for CM-pGFP::CM, solid upside down triangle for CM-pgp5S, solid diamond for CM-Δpgp5 and open circle for CM-Δpgp7). Note that mice infected with CM-pgp5S or CM-Δpgp5 but not CM-Δpgp7 developed significantly lower levels of inflammatory infiltration. *p<0.05 while **p<0.01 (Wilcoxon rank-sum test, all groups were compared against the full plasmid-complemented CM-pGFP::CM).

We next monitored the inflammatory infiltration in the oviduct tissue (Fig 7). As expected, the plasmid-free *C*. *muridarum* induced a minimal level of inflammatory cell infiltration in the oviduct tissues 60 day after infection. Interestingly, both versions of the Pgp5-deficient *C*. *muridarum* also induced significantly fewer inflammatory infiltration in the oviduct tissue, with the inflammatory severity scores of 3.33 ± 1.12 for CM-pgp5S (p<0.01) and 3.33 ± 1.52 for CM-Δpgp5 (p<0.05) respectively while the CM-pGFP::CM organisms induced an inflammatory severity score of 6.27 ± 0.60. We further compared the cytokine levels in oviduct tissue homogenates harvested on day 10 from mice infected with CM-pGFP::CM or CM-pgp5S (Table 1). We found that 20 out of the 27 cytokines measured were significantly lower in the oviducts of mice infected with CM-pgp5S than those with CM-pGFP::CM. Many of these reduced cytokines in the CM-pgp5S-infected oviduct tissues were pro-inflammatory cytokines such as IL-6, IL-12, IL-15, IL-17 & IL-18 and chemokines such as KC, MIP-1a & b, MIG as well as growth factor such as FGF and M-CSF. It is clear that CM-pgp5S is attenuated in inducing inflammation in the mouse oviducts.

**Table 1:** Cytokines from oviduct tissues of mice infected with *C*. *muridarum* with or without Pgp5 deficiency.

Cytokine	CM-pGFP::CM (pg/ml, n = 5)	CM-pgp5S (pg/ml, n = 5)	Ratio (CM-pGFP::CM/CM-pgp5S)	*P* value (*t* test)
IL-1α	676.96±712.06	47.69±65.34	14.20	0.13
IL-1β	1361.33±1037.16	100.69±126.13	13.52	0.06
IL-3	1.03±1.09	0.2±0.4	5.13	0.21
IL-4	11.67±1.2	0.99±1.2	11.79	**0.01**
IL-5	8.23±1.45	2.41±3.72	3.41	**0.05**
IL-6	247.35±88.29	0.72±1.42	344.73	**0.01**
IL-10	35.8±1.26	10.55±6.02	3.39	**0.01**
IL-12 (p40)	260.56±7.13	99.52±46.75	2.62	**0.01**
IL-12 (p70)	76.02±52.87	6.39±12.78	11.90	**0.05**
IL-13	842.2±210.28	231.43±184.18	3.64	**0.01**
IL-17	15.54±4.35	5.36±2.69	2.90	**0.01**
Eotaxin	693.49±606.1	0±0	n/a	0.06
G-CSF	2861.4±1212.6	248±58.61	11.54	**0.01**
IFN-γ	88.59±24.11	17.1±34.2	5.18	**0.03**
KC	356.65±247.23	28.18±40.11	12.66	**0.04**
MCP-1	5451.55±2491.63	1542.87±1687.02	3.53	**0.05**
MIP-1α	266.7±46.11	83.09±112.67	3.21	**0.05**
MIP-1β	112.71±34.37	28.98±11.06	3.89	**0.01**
RANTES	635.42±134.27	398.41±371.05	1.59	0.35
IL-15	87.04±13.29	0	n/a	**0.01**
IL-18	25.26±15.03	0	n/a	**0.02**
FGF-basic	1441.12±313.81	753.73±182.01	1.91	**0.01**
LIF	167.25±31.97	24.54±22.88	6.81	**0.01**
M-CSF	375.22±144.59	76.84±34.54	4.88	**0.01**
MIG	69415.57±7258.98	14064.71±14622.69	4.94	**0.01**
MIP-2	1457.69±1601.27	62.59±88.94	23.29	0.21
VEGF	1918.78±1275.58	842.52±345.24	2.28	0.16

Oviduct tissue homogenates were produced from mice infected with CM-pGFP::CM (2^nd^ column, n = 5) or CM-pgp5S (3^rd^ column, n = 5) on day 10 after intravaginal inoculation for simultaneously measuring 27 cytokines using a multiplex bead array assay. All cytokines are expressed in pg/mL as mean plus/minus standard deviation. The means were used for calculating ratio (2^nd^ last column) and Student’s t-test (last column). P values equal to or under 0.05 were listed in bold face. Note that 20 of the 27 cytokines were significantly lower in the oviducts of mice infected with CM-pgp5S than with CM-pGFP::CM.

## Discussion

Following our previous determination of Pgp3 as a key virulence factor in *C*. *muridarum* induction of hydrosalpinx, we have now presented evidence for an important role of Pgp5 in *C*. *muridarum* pathogenesis. First, Deficiency in Pgp5 via either pgp5 gene deletion or premature stop codon insertion significantly reduced the incidence and severity of hydrosalpinx induced by *C*. *muridarum*. The gross pathology evaluation was confirmed under microcopy. The pathogenicity attenuation effect is Pgp5-specific since deficiency in Pgp7 did not reduce *C*. *muridarum* pathogenicity. Second, although deficiency in Pgp5 did not affect the overall shedding course, the ascension to or survival in the oviduct tissue of the Pgp5-deficient organisms was significantly reduced on day 10 after infection. Interestingly, the organism did not show any defect in the endometrial epithelial tissue, suggesting that Pgp5 may selectively play a role in *C*. *muridarum* invasion of oviduct epithelial cells. Third, the reduced level of live organisms in the oviduct also correlated with the lower levels of inflammatory infiltration and cytokine production. Although it is not clear whether the reduced oviduct inflammation is due to the lower level of oviduct infection or reduced stimulation by the Pgp5-deficient organisms, we conclude that Pgp5 may promote *C*. *muridarum* pathogenicity in the upper genital tract by impacting both ascending infection and oviduct inflammatory responses.

We have previously shown that Pgp5 is a negative regulator [26]. Pgp5-deficient *C*. *muridarum* increased expression of some plasmid-dependent chromosomal genes that are normally positively regulated by Pgp4 [19,20,26]. However, it remains unknown how Pgp5 represses chlamydial chromosomal gene expression. Pgp5 is predicted to be a MinD. The chlamydial chromosome also encodes a MinD that may be required for chromosomal segregation. There is only ~30% homology between chromosomal MinD and Pgp5, suggesting that the genomic minD and the plasmid-encoded Pgp5 may have diverged enough from each other. The plasmid-encoded Pgp5 may have evolved additional functions different from MinD, for example, as a repressor for regulating gene expression in the chromosome. However, the Pgp5-mediated negative regulation is not required for *C*. *muridarum* to grow in cell cultures, suggesting that the Pgp5 function may be preserved for *Chlamydia* to survive in animals.

Since the plasmid is required for *C*. *muridarum* induction of hydrosalpinx at least in some strains of mice [6,7], we expected the Pgp5 deficiency to have an enhancing effect on the pathogenicity of *C*. *muridarum*. On the contrary, Pgp5 deficiency attenuated the pathogenicity of *C*. *muridarum*. How does the deficiency of the negative regulator Pgp5 attenuate pathogenicity? One possibility is that some of the Pgp5-repressed chromosomal genes may be able to suppress chlamydial growth in mouse oviduct epithelial cells. When Pgp5 is removed, these genes are optimally expressed and the gene products can then exert their suppression function. As a result, there is less inflammation in the oviduct, leading to the reduced hydrosalpinx. The result that significantly fewer live organisms were harvested from the oviduct tissue of mice infected with Pgp5-deficient *C*. *muridarum* seems to support this hypothesis. Alternatively, Pgp5 protein itself may be an inflammatory stimulator. The removal of Pgp5 could directly reduce the oviduct inflammation, which may partially explain the observation that the levels of 20 of the 27 cytokines measured were significantly lower in Pgp5-deficient *C*. *muridarum*-infected mice than the mice infected with the intact plasmid-transformed *C*. *muridarum*. We have recently shown that both the host complement factor C5 [35] and TNFR1 [10] play important roles in *C*. *muridarum* induction of hydrosalpinx. The question is whether Pgp5 plays any role in activating C5 or TNFR1 signaling pathways. In the end, regardless of how Pgp5 aids in *C*. *muridarum* pathogenicity, the fact that Pgp5 removal can significantly reduce the *C*. *muridarum* induction of hydrosalpinx suggests that Pgp5 can be a target for attenuating chlamydial pathogenicity.
